# Reinfection with a Bacterial Pathogen Augments Heterogeneity in Host Disease Responses

**DOI:** 10.21203/rs.3.rs-6856045/v1

**Published:** 2025-07-03

**Authors:** Jesse Garrett-Larsen, Anna Pérez-Umphrey, Arietta Fleming-Davies, James Adelman, Lauren Childs, Steven Geary, Kate Langwig, Dana Hawley

**Affiliations:** Virginia Tech; Virginia Tech; University of California, San Diego; University of Memphis; Virginia Tech; University of Connecticut; Virginia Tech; Virginia Tech

## Abstract

Individual responses to infection are often highly heterogeneous within host populations, with key consequences for transmission dynamics and pathogen evolution. Because re-exposure to pathogens is ubiquitous, understanding how priming exposures to a given pathogen alter inter-individual heterogeneity in immune responses and transmission-relevant traits is critical. Recent work in house finches (*Haemorhous mexicanus*) found that priming exposures to the bacterial pathogen *Mycoplasma gallisepticum* augment population-level heterogeneity in susceptibility to infection. However, it remains unclear whether priming exposures exacerbate heterogeneity in both underlying immune responses and transmission-relevant traits during reinfection. Using wild-caught, pathogen-naïve house finches, we experimentally tested whether priming with a low or high dose of *Mycoplasma gallisepticum* affects population-level heterogeneity in antibody responses, pathogen loads, and disease responses upon reinfection. We find that any prior exposure results in more heterogeneous antibody responses, and more variable pathogen loads upon reinfection. Further, the detected patterns in antibody variability with prior exposure match previously documented patterns of population-level heterogeneity in susceptibility upon secondary challenge, suggesting that variability in antibody responses within a population can be a relevant proxy for heterogeneity in susceptibility. Overall, we show that prior exposure to pathogens contributes to subsequent heterogeneity in transmission-relevant traits, with implications for downstream infection dynamics.

## Introduction

Host populations are rarely uniform in their responses to pathogen infection [1]. Instead, populations can fall along a continuum from homogeneity, such that all individuals within a population respond similarly to the same pathogen, to complete heterogeneity, such that some hosts are completely resistant to infection while others are wholly susceptible (often termed all-or-nothing responses [2]). This inter-individual variation, or population-level heterogeneity, can arise from innate differences within a population or from variable environmental conditions [3]. Regardless of the source, the heterogeneity of traits that mediate between-host dynamics, such as susceptibility (the probability of infection given exposure [2]), or infectiousness (the number of individuals infected by one infected host [4, 5]) can have a significant effect on epidemiological outcomes [4, 6–8] and host-pathogen evolution [9–11]. As a result, there is an increasing recognition of the utility of quantifying heterogeneity in traits alongside the traditional measures of central tendencies [21, 22]. While substantial work has characterized heterogeneity in between-host processes (e.g., infectiousness, contact rates, susceptibility) and their epidemiological effects [4, 5, 7, 12–16], there is still an incomplete understanding of how ecological factors such as prior exposure to pathogens contribute to the degree of inter-individual variation observed in processes that determine pathogen spread.

One important potential source of inter-individual heterogeneity in host populations is pathogen exposure history [12]. Individuals often vary significantly in infection-induced immune responses [23], and the protection that prior exposure confers is often incomplete or wanes over time [19, 24]. Recent work has shown that both vaccination [14] and prior exposure to a pathogen [13] can significantly increase the population-level variance in susceptibility, while simultaneously reducing a population’s overall mean susceptibility to reinfection. Further, the degree to which prior exposure augments heterogeneity in susceptibility may also depend upon the initial exposure dose [13], particularly if low dose exposures produce less complete protection against reinfection than do higher priming doses [25]. Experimental studies often use high exposure doses to elicit replicable responses [25], but, in nature, the exposure dose hosts experience is more likely to vary widely, and often fall in the lower range [17, 26]. However, it remains unclear whether exposure to low or high priming doses of pathogen induce underlying heterogeneity in immunological responses and subsequent responses to infection. Understanding how pathogen exposure history, a relevant source of variation in most disease systems, influences the degree of population-level heterogeneity is critical because of the documented consequences of such heterogeneity for outbreak risk [4, 15].

House finches (*Haemorhous mexicanus*) and their bacterial pathogen *Mycoplasma gallisepticum* (MG) are a well-characterized wildlife disease system that is well suited to address these questions. House finches are ubiquitous songbirds found across the United States and parts of Canada and Mexico. In the early 1990s, MG jumped from poultry into eastern U.S. house finches, triggering an epizootic that caused substantial population declines [27]. MG infections cause mild to severe inflammation of the conjunctiva (conjunctivitis), and mortality is likely due to reduced anti-predator responses in infected birds [28]. Today, MG is endemic in almost all free-living house finch populations [29] with seasonal outbreaks [30]. Pathogen load in the conjunctiva and disease severity are closely related in this system and serve as relevant proxies of transmission potential [29, 31–33]. Transmission occurs primarily via the shedding of MG from the conjunctiva of infected birds onto fomites (i.e., bird feeders), which is then spread to conspecifics [34, 35]. The viability of MG on fomites depends heavily on ambient conditions [36]. For these reasons, under natural conditions, house finches experience variable degrees and frequency of MG exposure. Further, while finches acquire protection from prior exposure to a range of pathogen doses [37], immune protection in this system is incomplete [13, 38], and reinfections are common [38, 39]. Recent work in this system demonstrated that prior exposure to MG augments population-level heterogeneity in susceptibility, and such heterogeneity in susceptibility can itself suppress pathogen outbreaks [13, 40].

**Figure 1:**
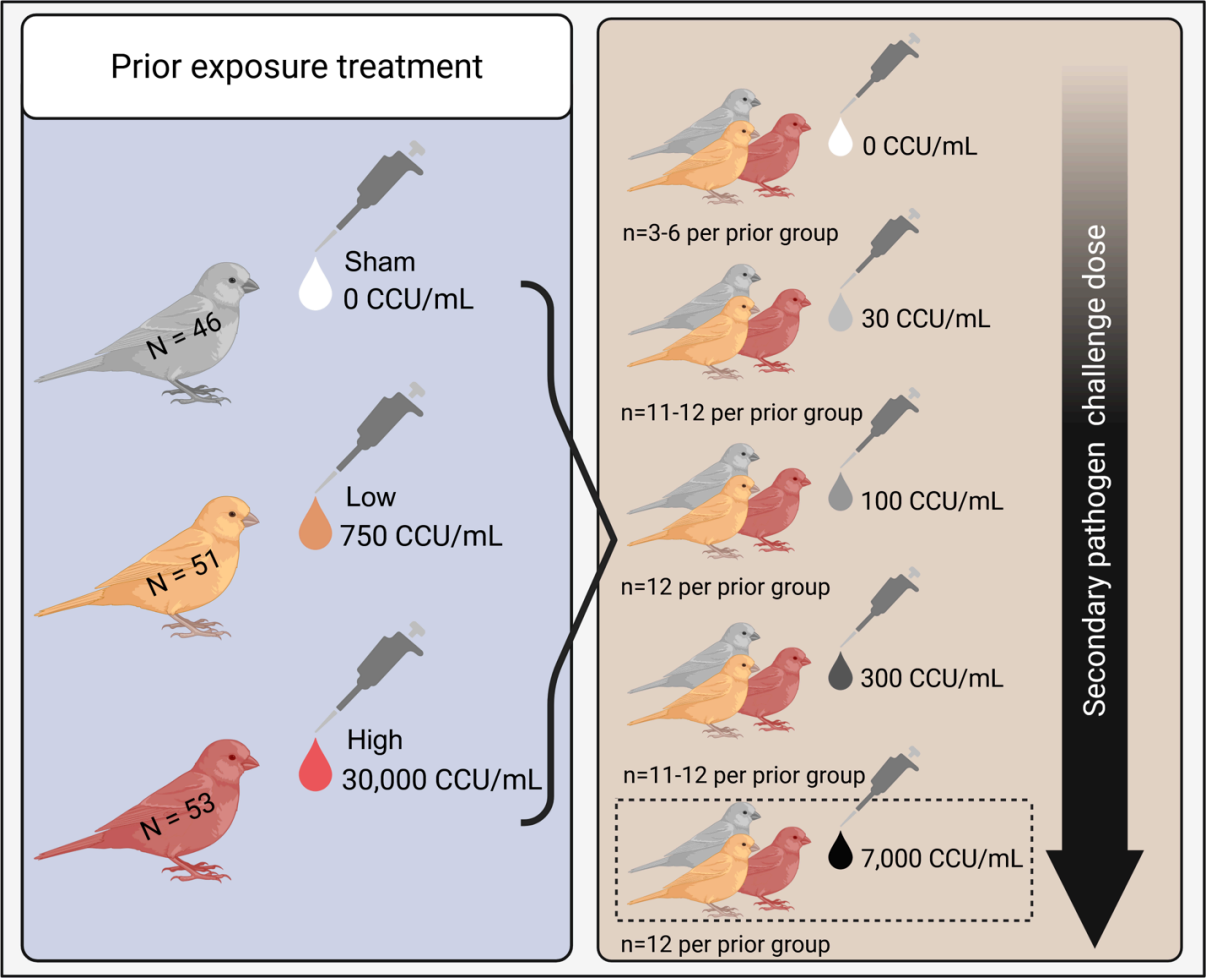
Experimental design to test how prior pathogen exposure influences host response heterogeneity upon secondary challenge. Birds were assigned one of three priming exposure treatment groups, receiving either a sham (white), low (orange), or high (red) dose of *Mycoplasma gallisepticum*. Birds were allowed to recover from their initial infection and then were re-challenged with one of five secondary challenge doses. While antibody analyses were done for all secondary doses, all analyses of variation in transmission-relevant traits (disease severity and pathogen load) were limited to birds given a high-dose secondary challenge only (7,000 CCU/mL; highlighted by dotted-line box) as this challenge dose produced the highest rates of successful reinfection, even within primed birds. Created in BioRender. Perez-Umphrey, A. (2025) https://BioRender.com/bxtgmef.

Here, we asked whether the extent of prior exposure to a pathogen alters population-level variation in host traits relevant to their infectiousness and susceptibility. We performed a two-part experiment (Fig. 1), where animals with experimentally varied pathogen exposure histories (i.e., none, low dose, or high dose priming) were then challenged with one of five secondary doses. Data were collected for a marker of the adaptive immune response (antibodies) just prior to secondary challenge, as well as two transmission-relevant traits, disease severity (pathology) and infectiousness (pathogen loads), in response to secondary challenge. These data were collected as part of a broader research effort to quantify heterogeneity in susceptibility, and so the overall experimental design and data collection process were the same as that reported in Hawley et al. [13]. However, in this study we analyzed distinct data from Hawley et al. [13], which focused solely on the proportion of birds infected (0|1) in each treatment group. Here, we analyzed heterogeneity in metrics of disease severity (pathology) and infectiousness (pathogen loads) following secondary pathogen challenge to ask whether prior exposure augments heterogeneity in disease responses upon reinfection. We also asked whether heterogeneity in acquired immunity (IgY antibodies) following priming pathogen exposure was associated with patterns of heterogeneity in susceptibility upon reinfection. We note here that, while the complexity of the immune system is not comprehensively represented by antibodies alone, they can be interpreted as a surrogate for broader immunological protection [41]. To maximize interpretability of our data, we picked a dual approach to quantifying inter-individual variation where we calculated both the Coefficient of Variation (CV) and a nonparametric alternative, Proportional Variability (PV) [42] for all traits. PV is nonparametric, independent from the mean, and bound between 0 and 1, allowing us to directly compare between traits on drastically different scales (e.g. pathogen load, eye score, and antibody levels). This enables us to robustly quantify variability while reducing biases that arise from small sample sizes and zero inflated data.

Overall, we predicted that prior pathogen exposure would augment heterogeneity in each trait measured relative to hosts that were naïve at the time of challenge. We also predicted that population-level heterogeneity in antibody levels at the end of the primary challenge would be positively associated with previously measured population-level heterogeneity in susceptibility in response to secondary challenge. Finally, given prior work [37], we predicted that prior exposure to MG would induce the greatest amount of heterogeneity in pathogen loads upon secondary challenge.

## Results

### MG Exposure Augments Both Mean and Heterogeneity in Antibody Responses

**Figure 2:**
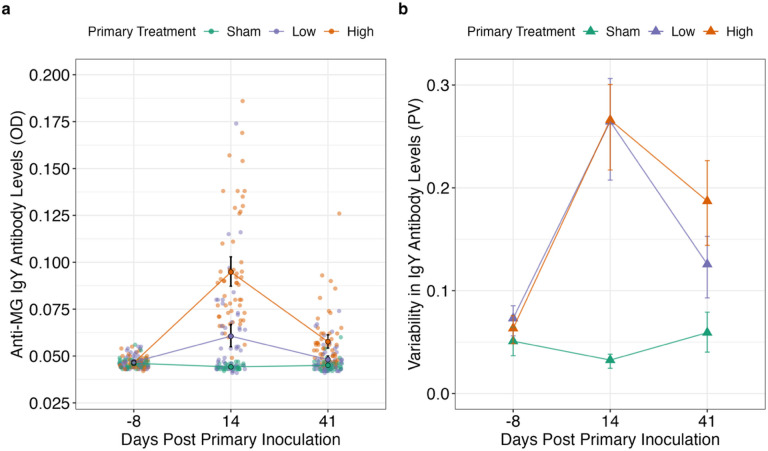
Antibody levels and their inter-individual variability in response to one of three priming exposure doses (sham control, low, or high) of *Mycoplasma gallisepticum*(MG). (a) Data points (translucent points) represent optical density (OD) antibody measures of individual birds, colored by primary treatment group (orange = “High”; purple = “Low”; green = “Sham”). Model predictions ± 2SEM (SE of the model) are represented by solid points and connecting lines. X-axis represents days post-priming inoculation. Y-axis represents anti-MG antibody levels as measured by OD. ELISA OD readings have a lower bound of 0.04. (b) Triangles connected by lines represent the calculated proportional variability (PV) of each group with 95% confidence intervals calculated by bootstrapping the data 1,000 times. X-axis represents days post-priming inoculation. Y-axis represents variability in anti-MG antibody levels as measured by OD.

First, we asked whether initial exposure to a pathogen (at low or high priming doses) augments inter-individual variation in antibody responses. We found that exposure to MG induced antibody responses that were both higher on average, and more variable among individuals within a given priming group. At both sampling points (here, 14- and 41-days post-priming inoculation [DPPI]), birds with exposure to MG at either priming dose (low or high) had significantly elevated antibody levels relative to controls (Fig. 2, Supplementary Table 1). However, the impact of priming dose on inter-individual antibody variability changed over time. Antibody responses were equally variable between the low and high priming dose groups 14 days post-priming (Low priming PV = 0.265; n = 50; High priming PV = 0.266; n = 53), whereas birds in the high-priming group harbored more variable antibody levels by day 41 post-priming than those in the low-dose or sham-inoculated groups (Sham priming PV = 0.059, n = 46; Low priming PV = 0.153, n = 50; High priming PV = 0.261, n = 53; High vs. Low: F = 5.68, p_adj_ = 0.019; High vs. Sham: F = 16.3, p_adj_ = 0.0003; Sham vs. Low: F = 7.75, p_adj_ = 0.0097; Fig. 2). CV was also calculated to allow for comparisons with previous work [43], and this metric indicated the same overall trends as PV in antibody data across the priming phase (Supplementary Fig. 1).

### Antibody Levels Predict Reinfection Probability

**Figure 3:**
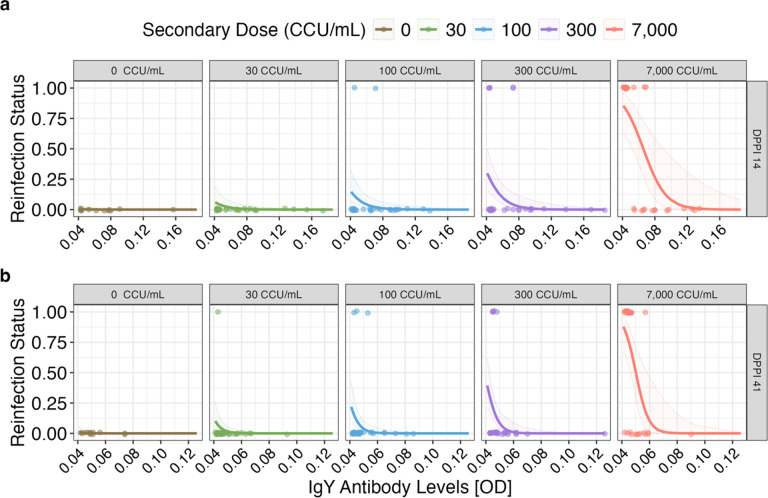
Individual variation in antibody levels after priming exposures to *Mycoplasma gallisepticum* (MG) predicts susceptibility to secondary challenge with one of five MG doses (facet labels), with reinfection status (y-axis) quantified as 0 (no) or 1 (yes). Model predictions (solid lines) ± 2SEM (SE of the model; shaded regions) of reinfection status (0|1; datapoints) for each secondary exposure dose on days post-priming inoculation (a) 14 and (b) 41. The x-axis represents antibody optical density [OD] values. Because secondary dose was an important predictor of reinfection likelihood, the five distinct secondary doses (facet labels) are shown separately.

Because antibody levels showed significant inter-individual variation in response to priming, we asked whether such variation was predictive of susceptibility to reinfection at the individual bird level. We used two independent generalized linear mixed effects models (Binomial distribution) across each post-priming sampling date (days 14 and 41 post-priming) to ask whether antibody levels generated in response to the priming phase of the experiment were predictive of susceptibility upon secondary challenge. Because birds received distinct secondary challenge doses, log_10_ secondary dose was included in the model. Antibody levels on both days post-priming 14 (*p* = 0.003, df = 128), and 41 (*p* = 0.003, df = 142), were predictive of susceptibility upon secondary challenge with MG, with birds with higher antibody levels having lower probability of infection upon secondary challenge (Supplementary Table 2; Fig. 3). As expected, the log_10_ secondary challenge dose was also predictive of susceptibility to reinfection, where birds challenged with a higher secondary dose were more likely to become reinfected regardless of days post-priming inoculation (DPPI 14: *p* < 0.001, df = 128; DPPI 41: *p* < 0.001, df = 142).

### Group-Level Variability in Antibody Levels Parallels Variability in Susceptibility

**Figure 4:**
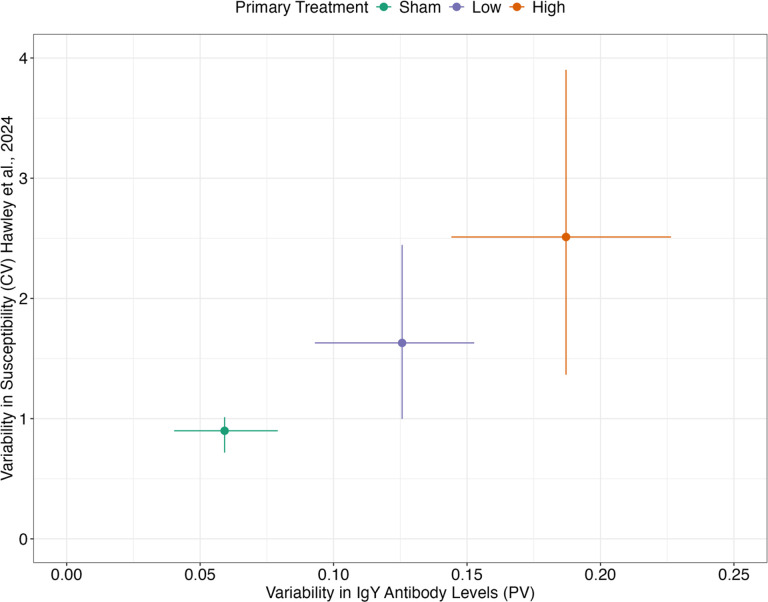
Group-level variability in antibody levels (x-axis) measured 41 days after inoculation with one of three priming pathogen doses (sham control, low, or high) qualitatively matches patterns in group-level heterogeneity in reinfection susceptibility (y-axis) upon secondary challenge on day 42 post-priming inoculation. Variability in reinfection susceptibility is shown as the gamma distributed coefficient of variation of susceptibility (CV; y-axis) reported in [15]. Variability in antibody levels the day prior to secondary challenge (x-axis) is calculated as proportional variability (PV). Error bars represent 95% CIs calculated by bootstrapping the raw data 1,000 times with replacement. Shapes are colored according to the primary treatment group (green: sham; purple: low; orange: high).

Next, we asked whether detected patterns in population-level immune heterogeneity with prior exposure (Fig. 2) matched patterns of heterogeneity in susceptibility observed in birds with prior MG exposure in previous work [13]. Indeed, the degree of variability (PV and CV) in antibody levels the day before reinfection challenge (DPPI 41) qualitatively matched the variability (CV) in susceptibility to reinfection following challenge on DPPI 42 (Fig. 4): birds that received a high priming treatment dose exhibited the greatest heterogeneity in antibody levels just before their secondary challenge (PV = 0.187; n = 53), and similarly showed the greatest heterogeneity in susceptibility to that challenge (CV = 2.511 as reported in Hawley et al. [13]). Birds without any prior exposure (i.e., received a sham control dose during primary challenge) responded more homogeneously (in terms of both antibody levels and susceptibility) to the same series of secondary challenge doses that revealed heterogeneity in birds that had been primed with either low or high treatment doses (Fig. 4).

### Priming Exposure Modulates Heterogeneity in Disease Response

**Figure 5:**
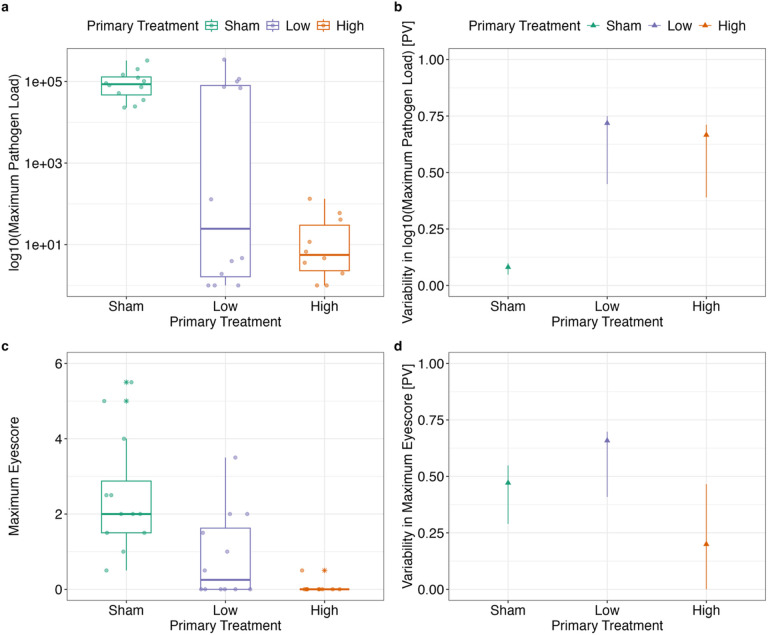
Maximum pathogen loads (a) and disease severity scores (c), as well as their group-level variability (b and d), in response to a high-dose pathogen challenge [7,000 CCU/mL] for birds given distinct prior exposure treatments (sham control, low, or high priming dose). Maximum (a) log10(pathogen load) and (c) eyescore per bird following secondary challenge. Datapoints represent all birds inoculated with the highest secondary dose of *Mycoplasma gallisepticum*. All other secondary dose groups were excluded as not enough individuals became reinfected to meaningfully quantify heterogeneity. Box plot whiskers extend to values ± 1.5x the IQR, center line represents the median, and solid, colored stars denote outliers. Variability in (b) maximum log10(pathogen load) and (d) eyescore (bottom right) following secondary challenge. Colored triangles show proportional variability (PV) of disease responses calculated per treatment group. Error bars represent 95% confidence intervals for the PV calculated by bootstrapping the data 1,000 times with replacement.

We next asked whether pathogen exposure history induces inter-individual heterogeneity in transmission-relevant metrics (pathogen load and disease severity) during reinfection. Here we limited our analyses to only the highest dose (7,000 CCU/mL) secondary challenge group, because other challenge doses resulted in low rates of reinfection (see Fig. 3), making it difficult to quantify heterogeneity in disease responses. Upon secondary challenge with a high dose of MG, birds with prior exposure to MG had more heterogeneous maximum log_10_ pathogen loads (Low priming PV = 0.72; High priming PV = 0.67) than birds with no prior exposure (Sham priming PV = 0.08; Fig. 5, Supplementary Table 3). Brown-Forsythe tests revealed that each priming treatment resulted in a statistically distinct level of variability in maximum log_10_ pathogen loads with the lowest prior exposure group showing the highest variability (Low vs. High: F = 11.99, p_adj_ = 0.0037; Low vs. Sham: F = 22.16, p_adj_ = 0.00032; High vs Sham: F = 4.95, p_adj_ = 0.038).

In contrast, heterogeneity in the maximum disease severity (measured here as eyescore; see methods) a bird exhibited post-secondary challenge was lowest in the high prior exposure group (Sham PV = 0.47; Low PV = 0.66; High PV = 0.2; Fig. 5, Supplementary Table 3). Brown-Forsythe tests showed significant differences between the high prior exposure group and both the low and sham priming exposure groups in variability in maximum eyescore, confirming this result (Low vs. High: F = 7.22, p_adj_ = 0.021; High vs. Sham: F = 7.45, p_adj_ = 0.021). However, there was not a significant difference in variability between the low and sham priming groups (F = 0.22, p_adj_ = 0.64).

### Prior Exposure Confers Protection to Secondary Challenge

Last, we confirmed that prior exposure to either low or high doses of MG conferred acquired protection against secondary challenge. Priming exposures of MG resulted in reduced susceptibility to reinfection (p = 0.0002; Secondary Infection Rate at 7,000 CCU/mL: High Priming = 2/10, Low Priming = 6/12, Sham Priming = 12/12; Supplementary Table 3). Additionally, birds with prior exposure showed significant reductions in maximum log_10_ pathogen loads (X^2^ = 13.604, df = 2, p = 0.001), and pathology (X^2^ = 19.06, df = 2, p < 0.0001). Post-hoc pairwise comparisons showed that prior exposure to either MG dose resulted in significantly lower maximum log_10_ pathogen loads (High vs. Sham: z = −3.59, p_adj_ = 0.0005; Low vs. Sham: z = −2.44, p_adj_ = 0.022; High vs. Low: z = −1.26, p_adj_ = 0.31) as well as significantly lower maximum eye scores (High vs. Sham: z = −4.30, p_adj_ < 0.0001; Low vs. Sham: z = −2.73, p_adj_ = 0.01, High vs. Low: z = −1.69, p_adj_ = 0.14) compared to birds with no prior exposure.

## Discussion

Quantifying population-level heterogeneity in traits relevant to host-pathogen interactions can present novel insights beyond those gained from mean values alone [44]. In particular, heterogeneity in host traits relevant to pathogen fitness can have key impacts on downstream epidemiological [4] and evolutionary [45] dynamics. Because host populations are typically composed of individuals with variable infection histories and acquired protection [23], it is important to understand how prior pathogen exposure modulates the degree of inter-individual heterogeneity in host traits relevant to immunological protection and pathogen spread. Here, we used a two-phase, dose-response experiment to determine whether pathogen exposure history induces heterogeneity in host disease traits relevant to infection probabilities and pathogen spread. Additionally, we were able to compare heterogeneity in a readily measurable immune marker of prior exposure to estimates of heterogeneity in susceptibility presented in previous work [13], but generated from the same birds used here. We show that, relative to a pathogen-naïve population (sham controls), hosts with prior exposure show significantly higher inter-individual heterogeneity in both an immunological trait relevant to susceptibility, and in a transmission-relevant trait (pathogen loads in response to secondary challenge). Further, the detected patterns of population-level heterogeneity in antibody levels in response to prior exposure correspond with previously documented patterns of heterogeneity in susceptibility in this system.

Our primary objective was to quantify whether prior exposure to pathogens alters the degree of inter-individual variation in host responses. We first examined a marker of immunological protection and found that initial exposure dose was positively associated with group-level variability in IgY anti-MG antibody levels directly before secondary challenge. In turn, this individual variation in antibody levels was predictive of reinfection probability, consistent with previous work in this system [39] (Fig. 3). This suggests that prior exposure to pathogens does not just alter the average level of protection in a population, but can also modulate the degree of variation in protection that is present, which can have key population-level impacts for disease spread [7, 13, 16]. Interestingly, we found that the degree to which prior exposure augmented variability in antibody responses at the later sampling timepoint (DPPI 41) depended on the dose of prior exposure, while the degree of variability in antibody responses was similar across prior exposure doses for the earlier timepoint (DPPI 14) representative of peak responses. This change in patterns of heterogeneity across groups appear to arise because birds inoculated with a high priming dose maintained higher mean levels of antibodies across post-priming sampling periods than those inoculated with a low dose. This suggests that the kinetics of antibody responses post-exposure may be important to account for when determining to what degree prior exposure to pathogens augments inter-individual variability in antibody responses. House finches acquire incomplete but significant protection from prior exposure to MG, and birds that are exposed to a higher priming dose generate higher mean antibody levels, as we show here and in prior work [22, 37]. But antibody levels wane over time in this system [36, 44, 45]. This combination of incomplete initial immunity, even from high priming doses [36], and waning immunity over short time scales may be a mechanism by which prior exposure generates heterogeneity in antibody responses and subsequent disease responses.

The ability to link easily measurable biomarkers to population-level measures of susceptibility and disease responses can help to broaden the use of models that incorporate heterogeneity in susceptibility to inform management decisions [48]. Pathogen-specific antibodies are a widely applicable, relevant, and convenient biomarker that have been used for decades as a proxy for estimating standing immune protection [49, 50]. Here, we add to the growing evidence that the magnitude and durability of antibody response is positively associated with prior exposure dose in this system [39, 51], and that antibody levels just prior to inoculation are predictive of protection from reinfection [38, 39]. Importantly, the primary dose that an individual received was intentionally not included in our models that compared antibody levels with reinfection probability (Supplementary Table 2). Therefore, without knowing the dose of pathogen that individuals were exposed to, we could still predict group level susceptibility to secondary challenge based solely on antibody levels that were collected after birds had resolved their infection.

Population-level heterogeneity in antibody levels has been studied in the context of the recent SARS-CoV-2 pandemic, showing significant variability in antibody titers between groups (age, sex, vaccination status) [49]. Importantly, in another study, within-group variability in antibody titers was associated with functional differences in immunity distinct from absolute antibody concentration. Specifically, individuals with maximum antibody titers above the group median exhibited qualitatively different functions (e.g. higher neutralizing antibody titers, antibody-dependent complement deposition, and antibody-dependent neutrophil phagocytosis) compared to those below the median [52]. Thus, it is important to not only measure central tendencies, but also variability, as focusing solely on these central tendencies obscure biologically important variation in immune function. Indeed, variability in antibody levels may be associated with variability in other aspects of acquired immunity through their effector functions (opsonization, Fc-mediated phagocytosis, antibody-dependent complement activation [52]), though not explicitly predictive of cellular responses [53].

While we did not directly measure antibody effector functions here, we were able to compare variation in antibody levels with that of previously measured functional variation in susceptibility, defined here as the likelihood of infection given a known dose of pathogen.

Population-level heterogeneity in susceptibility is measured through resource intensive dose-response experiments [13, 14, 44, 54]. These experiments are valuable in that they allow for an assessment of variability in susceptibility that is independent of the population’s mean [44], and epidemiological models that account for such heterogeneity in susceptibility more accurately predict outbreak size [7]. However, these dose-response experiments and subsequent quantification of heterogeneity in susceptibility are not typically feasible with field-collected data where exposure dose is difficult or impossible to determine and likely highly variable. Because we found that prior exposure history produced parallel effects on heterogeneity in antibody levels and previously measured heterogeneity in susceptibility in this system [13], measures of antibody heterogeneity from field-sampled birds could be used as a proxy for heterogeneity in susceptibility, allowing models to more accurately predict epidemic size without intensive dose-response experiments. Future work could test the utility of this idea by creating Susceptible-Infected-Recovered (SIR) epidemic models where susceptibility varies continuously according to a standardized distribution, which is parameterized from dose-response experimental data, e.g. [15, 16]. Then, these results could be compared to an alternative SIR model where a continuous distribution of susceptibility is still incorporated but is parameterized by the empirical distribution of IgY antibody levels.

The second main goal of our study was to understand whether prior exposure to MG augmented heterogeneity in traits relevant to transmission, such as pathogen loads. To test this, recovered individuals were re-challenged with one of five secondary doses, with our analyses here limited to the highest secondary dose where reinfections were more likely. We found that both low and high dose prior exposure led to higher inter-individual variation in log_10_ maximum pathogen load upon reinfection, relative to birds with no prior exposure. Interestingly, while both the PV and CV metrics indicate relatively similar amounts of variability between low and high dose prior exposure groups in log_10_ maximum pathogen load upon secondary infection (PV_low_ = 0.72, CV_low_ = 1.00; PV_high_ = 0.67, CV_high_ = 0.83), the orders of magnitude differ substantially (range_low_ = 0–5.54; range_high_ = 0–2.12; Fig. 5). This suggests that the variability present within the low-dose priming group may be more biologically meaningful for pathogen spread, which tends to be highly dependent on pathogen loads above ~ log_10_ of 4 in this system [37]. The similarity of PV values for pathogen loads between the high and low dose groups, despite large differences in order of magnitude of the load ranges, also points to potential constraints with using the PV as a variability metric. While convenient for comparisons across different disease response traits, PV can be difficult to interpret in situations where groups vary in orders of magnitude within one trait (e.g. pathogen load). Close examination of the raw data is important in these situations.

We also examined whether prior exposure altered inter-individual heterogeneity in maximum disease severity. Although priming with a low or high dose led to a significant decrease in maximum pathology upon reinfection as expected, there were no clear effects of prior exposure on variability in maximum disease severity. House finches in Virginia, the source population for the birds used in our experiment, have evolved tolerance to infection [29] (minimizing pathology per pathogen load). This tolerance may have reduced the range and maximum disease severity expressed within our host population, potentially limiting our ability to detect effects of prior exposure on inter-individual variability in disease severity. On the other hand, because prior exposure or vaccination can produce stronger protection against disease versus infection load in this system [22] and others e.g. [53], it is possible that acquired protection generated from prior exposure, particularly to high priming doses, generally reduces inter-individual variation by producing more complete disease protection.

Overall, we found that prior exposure to MG augments variability in both antibody responses and in infection loads during reinfection. Because pathogen loads during reinfection were shown to predict the likelihood of spread to a naive cagemate [37], increased variation in loads among individuals during reinfection should further reduce subsequent outbreak size, along with reductions in mean susceptibility and increases in variation in susceptibility [13]. While past studies considered heterogeneity in transmission more broadly [4] and in pathogen loads in particular [55–57], future work should investigate specifically how heterogeneity in traits such as pathogen loads affect pathogen spread, as this heterogeneity may represent an important yet overlooked additional layer of protection from subsequent outbreaks. Practically, because we found that inter-individual heterogeneity in antibody responses matched patterns for heterogeneity in susceptibility, our results also suggest that quantification of pathogen-specific antibodies may be a rapid and easy way to estimate population-level heterogeneity in susceptibility in some systems. Ultimately, such approaches may allow easier incorporation of variation in infection-induced immunity into mathematical models [23], as researchers are increasingly doing [58, 59]. Given that many host-pathogen systems, including those of humans, are characterized by incomplete acquired protection [60–62], understanding how prior exposure alters the degree of variation among hosts is critical for predicting both outbreak dynamics and the selection pressures acting on pathogens in populations of hosts with variable exposure histories.

## Methods

### Ethics Statement

All bird handling, husbandry, and experiments were approved by the Virginia Tech IACUC (protocol #21–082). Capture and collection of house finches was approved by the Virginia Department of Game and Inland Fisheries (066646) and USFWS (MB158404). Authors complied with ARRIVE guidelines.

### Experimental design

To ask whether prior pathogen exposure history determines the degree of variation in host traits relevant to transmission dynamics (antibody responses, pathology, and pathogen loads), we designed a fully factorial 3 × 5 two-phase experiment, where we first experimentally varied host prior pathogen exposure, allowed for complete infection recovery (forty-two days), and then re-exposed birds using a gradient of secondary challenge doses. Free-living, hatch-year house finches were captured and confirmed to be pathogen-naive (n = 155; see Supplementary Methods for capture and quarantine details) prior to assignment to one of three primary treatments (exposure doses: sham [n = 51], low [n = 51], or high [n = 53]). For secondary challenge, birds from each primary treatment group were assigned one of five secondary exposure doses (Fig. 1). Birds were stratified by sex and randomly assigned to priming and secondary treatment groups within sex to ensure equal sex ratios between groups.

### Sample & data collection

#### MG IgY Antibodies

We collected blood samples (~ 80 μL) from each bird by brachial vein puncture with a sterile 26-gauge needle. We collected blood using heparinized capillary tubes which was then expelled into 1.5 mL centrifuge tubes and kept on ice until centrifugation (13,000 × RPM for 7 minutes) to separate plasma from red blood cells. Plasma samples were stored at −20°C until use in ELISA assays. We used a commercially available ELISA kit (FlockChek M. gallisepticum antibody enzyme-linked immunosorbent assay kit [IDEXX, Wesbrook, Maine, USA]) to measure house finch anti-MG IgY antibodies from serum samples. We followed the protocol outlined in Hawley et al. [63], using a 0.061 OD (optical density) seropositivity threshold cutoff. Sixteen plasma samples were lost during processing (see Supplemental Methods). Importantly, fourteen of these were from sham inoculated birds which would have been seronegative, having never been exposed to MG. Had these fourteen individuals been included in our logistic regressions, our effect sizes would be even more pronounced.

### Pathology

We assessed clinical status according to eye lesion severity, which was scored on a 3-point scale in 0.5 increments (0 = no detectable swelling or inflammation; 1 = minor swelling around the ring of the eye; 2 = moderate swelling and eversion of the conjunctival tissue; 3 = eye nearly hidden by swelling and crusted exudate [47]), and summed across both eyes for a composite score. The same experienced observer collected these data while blind to a given bird’s treatment group to eliminate observer-bias.

### Pathogen Load

Following eye scoring, we swabbed the conjunctiva of each eye with a sterile cotton tipped applicator and then vigorously swirled in tryptose phosphate broth (TPB) for 5 seconds. The left and right eyes of each bird were swabbed separately and then pooled into a tube containing 0.3 mL TPB. Swabs were discarded and the sample solution was kept on ice and stored at −20°C until DNA extraction. We extracted MG DNA from the TPB solution using 96-well Qiagen DNeasy Blood and Tissue Kits (Qiagen, Hilden, Germany). Pathogen load was quantified via quantitative real-time PCR (qPCR; QuantiNova Kit [Qiagen, Hilden, Germany]) on a QuantStudio 5 using probes, primers, and plasmid standards with *mgc2* gene insert [63, 64].

Pathogen loads were analyzed as log_10_(pathogen load copies + 1; to account for zeros in the dataset). This is referred to simply as log_10_ pathogen load in this text. For analyses relating antibody levels or prior exposure treatment to reinfection susceptibility, we conservatively defined infection as > 50 MG pathogen copies at any time point post-secondary challenge [13]. Because birds may be asymptomatically infected [65], pathology alone was not used to define infection status (sample sizes: Supplementary Methods; Supplementary Table 4).

### Data analysis

All data were analyzed in RStudio (v4. 3. 1; R Core Team 2023). Model selection was performed using Akaike’s Information Criterion adjusted for small sample sizes (AICc [66]).

### Measures of heterogeneity

We estimated inter-individual heterogeneity of all measures of interest (antibody levels, pathogen load, and disease severity) using proportional variability (PV), a measure of variation within groups that is independent of the mean [42, 67]. In contrast to Pearson’s coefficient of variation (CV), PV is less sensitive to rare events, does not assume a normal distribution, and, importantly for our dataset, is not biased by zero-inflation and nonparametric data [68]. Initially, CV was preferred as it accounts for the tendency of variance to increase with the mean. Recent work has suggested that the CV introduces bias when rare events are included or the mean approaches zero, therefore, several new approaches to calculate dispersion independently of measures of central tendencies have been developed [42, 67, 69–71]. The PV is useful for comparisons of variability between measurements on different scales as it is a true proportion bound between 0 and 1 and it accounts for strongly disparate patterns exhibited by different groups [72]. This metric is particularly suited to quantify the degree of inter-individual variation in continuous host traits, such as antibody levels or pathogen loads. In contrast, PV is not suited to measure variability in binomial data such as infection status or susceptibility, as in these cases variability is inherently tied to the mean. We calculated PV using the R package *CValternatives* [73]. We also calculated CV for some analyses in order to draw comparisons with previous analyses [13], and contrast with PV to robustly quantify variability.

To compare measures of variability between groups and over time, we estimated 95% confidence intervals by manually bootstrapping the raw data 1,000 times per group with replacement. We used the 2.5th and 97.5th percentiles of the distribution of the bootstrapped data to estimate the 95% confidence intervals for each variability metric. Additionally, Brown-Forsythe tests, which compare the amount of absolute deviation from the median within each group [74], were used to assess differences in variability across treatment groups. Importantly, the Brown-Forsythe test was developed as an alternative to Levene’s test to be robust to non-normal distributions [75]. Post-hoc analyses were then performed using pairwise Brown-Forsythe tests with Benjamini-Hochberg corrections for multiple comparisons.

### Variability in host responses

We first asked whether prior exposure induced differences in the mean or heterogeneity in antibody levels in a degree-dependent manner (none, low, or high prior exposure), and whether this heterogeneity matched documented patterns of heterogeneity in susceptibility during a secondary challenge [13]. Variability metrics (PV and CV) were calculated for antibody levels within each primary treatment group per day (DPPIs – 8, 14, and 41) over the primary phase of the experiment. We then asked whether individual variability in antibody levels during the priming phase (measured at two distinct time points) were predictive of reinfection susceptibility. We used a generalized linear mixed effects model (GLMM; glmmTMB [76]) with a Gamma distribution and log link to test whether the interacting fixed effects of primary inoculation dose (categorical: none, low, or high) or days since primary inoculation (DPPI; factor with levels – 8, 14, and 41) predicted IgY anti-MG antibody levels (reported as ELISA optical density [OD] values). Individual bird identity was included as a random effect to account for repeated measures. Additionally, dispersion was modeled as a function of DPPI and primary treatment. Model selection was performed using Akaike’s Information Criterion (AICc; aictab, *bblme*). The model was fit using maximum likelihood estimation, with significance assessed using Wald z-tests with a significance threshold of α = 0.05. After fitting the model, we conducted post-hoc pairwise comparisons to explore the effects of different primary inoculation doses on each day post inoculation. Comparisons were performed using Tukey’s adjustment for multiple comparisons (emmeans [77]), to determine differences in estimated marginal means at each day post inoculation.

To ask whether there was a relationship between IgY antibody levels during the priming phase and susceptibility to reinfection in the secondary phase, we fit two independent GLMMs with binomial distributions and a logit link for DPPIs 14 and 41. We asked whether antibody levels, secondary dose (transformed as log_10_ + 1; referred to as log_10_ secondary dose), or their interaction were predictive of reinfection susceptibility. No significant interaction was evident between antibody levels and log_10_ secondary dose and therefore the interaction term was removed from the models. Likewise, bird sex was not predictive of antibody levels or susceptibility upon reinfection and was therefore excluded from the final models. Models were fit using maximum likelihood estimation. Significance was assessed using Wald z-tests with a significance threshold of α = 0.05. Model selection was performed using Akaike’s information criterion (AICc) values.

The second part of our analyses focused on whether prior exposure induces inter-individual variability in traits relevant to infectiousness (eye score and pathogen load). As part of the second phase of our experiment, birds were re-challenged with one of five secondary doses. Due to the protective effects of prior exposure, there were few successful reinfections in the four lowest secondary doses, and so we limited our analyses to those birds that received a high secondary dose (7,000 CCU/mL) of which there were successful reinfections (n = 21/34).

While not all individuals re-challenged with the 7,000 dose became reinfected, we included all individuals in our analyses, regardless of reinfection status to maintain all biologically relevant variability and unobserved heterogeneities [78]. We tested whether infection rate (1|0) was dependent on priming dose using Fisher’s Exact test [79]. Post-hoc pairwise Fisher’s Exact tests were then performed with a Holm adjustment for multiple comparisons. PVs were calculated per treatment group using each bird’s maximum eyescore and maximum log_10_ pathogen load during secondary challenge. We also tested for mean differences in maximum eyescore using a Kruskal-Wallis test [80], followed by a pairwise Dunn’s test with Bonferroni adjustments [81]. Pathogen load is generally log_10_ transformed in this disease system [64]. This scaling emphasizes the relative biological importance of the orders of magnitude and highlights the differences between individuals with low and high pathogen loads. However, transformations change the shape of the data and can affect the quantification of variability by augmenting variation at low values and compacting it at high values. Therefore, while we present our pathogen load data as log_10_-transformed in the main text, we have included variability metrics for both log_10_ transformed and untransformed pathogen load data in the Supplement for comparison (Supplementary Table 5, Supplementary Fig. 2). Importantly, using log_10_ transformed pathogen load did not affect variability metrics for groups with prior exposure. Transforming the data does, however, reduce the CV and PV compared to the raw pathogen load in the control birds that were subsequently inoculated with 7,000 CCU/mL MG.

## Supplementary Files

This is a list of supplementary files associated with this preprint. Click to download.

- ReinfectionAugmentsHeterogeneitySupplement.docx

## Data Availability

The datasets and code are publicly available at https://github.com/jessegl97/EEID_1A_Mechanistic_Link/tree/d5c9b8da7173f4eda08112544f07c7bcdb6bee41/Reinfection_Augments_Heterogeneity/Public.
